# Therapeutic effects of *Byrsocarpus coccineus* root bark extract on bacterially and chemically induced diarrhea in the Wistar albino rat (*Rattus norvegicus domestica*)

**DOI:** 10.1002/ame2.12094

**Published:** 2019-12-18

**Authors:** Ejeh Augustine Sunday, Patrick Azubuike Onyeyili, Saganuwan Alhaji Saganuwan

**Affiliations:** ^1^ Department of Veterinary Physiology and Biochemistry Faculty of Veterinary Medicine University of Abuja FCT Nigeria; ^2^ Department of Veterinary Pharmacology and Toxicology College of Veterinary Medicine Federal University of Agriculture Makurdi Benue State Nigeria

**Keywords:** antidiarrhoea, *Bryocarpus coccineus*, castor oil, diarrhea, *E. coli*, extract, hematonic, rat

## Abstract

**Background:**

Diarrhea can be caused by pathogenic microorganisms and chemicals. In view of this, *Byrsocarpus coccineus* Schum and Thonn (Connaraceae) was used to treat diarrhea induced by castor oil or bacteria in Wistar albino rats.

**Methods:**

Qualitative and quantitative analyses of an aqueous root back extract of *B. coccineus* were made and the acute toxicity, antidiarrhea properties, and in vitro and in vivo antimicrobial activities of the extract were investigated in rats.

**Results:**

The phytochemical analysis of the root bark extract revealed the presence of flavonoids, alkaloid, saponins, tannins, and phenols. The quantitative analysis showed that saponins formed 10.6% of the extract, tannins 7.6%, flavonoids 6.2%, phenol 5.8% and alkaloids 4.4%. A dose limit of 5000 mg/kg was safe to use in the rats. At a dose of 100 mg/kg, the extract decreased distance travelled by activated charcoal in the gastrointestinal tract, frequency of defecation, and number of unformed faeces caused by castor oil‐induced diarrhea, and led to 74.96% inhibition of the diarrhea effects. *Escherichia coli* and *Salmonella pullorum* were susceptible to higher concentrations of the extract with a minimum inhibitory concentration of 0.3125 mg/mL. *E. coli*‐infected rats showed depression, weight loss, anorexia, diarrhea, and weakness, which was ameliorated by the extract on day 2 post treatment. Observed congestion, cellular infiltration and necrosis of the liver, intestine and kidney following infection were improved by the extract.

**Conclusion:**

*B. coccineus* extract can be used in the treatment of anaemia, and castor oil‐ and *E. coli*‐induced diarrhea in rats.

## 1 INTRODUCTION

Diarrhea is defined as an excessive fluid loss of 200 g (200 mL) per day.^1^ Diarrhea diseases cause several million deaths in the world annually, and diarrhea is the most common cause of morbidity and mortality globally, affecting many infants in the developing countries.^2^, ^3^, ^4^ Overcrowding, poor sanitary conditions, contaminated water, inadequate food hygiene and poor nutrition predispose mainly children to the risk of diarrhea diseases.^5^, ^6^ Ingested food components or medicines such as artificial sweeteners and lactose may also result in diarrhea*.* 7 Evaluating the risk of diarrhea diseases requires knowledge of the complex interaction between biological, socio‐economic, behavioral, and environmental factors.^8^ In Nigeria, for example, the Nigeria Demographic and Health Survey reported that diarrhea prevalence is highest in the northeast (22%) and lowest in the southwest (7%).^9^ Diarrhea induces dehydration and electrolyte imbalance, especially in children, infants and frail elderly patients.^10^ Acute watery diarrhea usually lasts for less than 7 days, although it can last for up to 14 days.^11^ Diarrhea is also caused by a wide range of bacteria such as *Campylobacter jejuni, Escherichia coli, Salmonella* species and *Aeromonas* species.^12^ Loss of large quantities of intestinal electrolytes and fluid secretion occurs in patients with cholera caused by *Vibrio cholera.* Cholera toxin activates adenylate cyclase by causing ADP‐ribosylation of the G‐proteins, resulting in constitutive stimulation of the cyclase.^13^ Elevated cAMP levels in turn activate protein kinase A and protein phosphorylation opens the luminal Cl‐channel in secretory cells and inhibits Na^+^/H^+^ exchange in absorptive cells. The net result is gross NaCl secretion. Management of diarrhea involves the use of rehydration therapy, nutritional supplements, drugs^14^ and plant extracts.^15^

Medicinal plants are the primary source of medication used as complementary or alternative treatments to orthodox medicine.^16^ *Byrsocarpus coccineus* Schum and Thonn is a climbing shrub found in west and central African countries including Nigeria, Ghana, Cameroun, Ivory Coast, Togo, Central African and Congo. It is known as Onyakpechi in Igala, Tsamiyar kasa in Hausa, Oke abolo and Mybo‐apapea in Yoruba and Onyakwachi in Idoma.^17^ It is used in traditional African medicine for the treatment of earache, gonorrhea, impotence, jaundice, piles, diarrhea, tumour and wounds,^18^ and has been shown to have anxiolytic, sedative,^19^ anti‐inflammatory,^20^ analgesic,^21^ antidiarrhea,^22^ antimicrobial^23^ and uterotonic activities.^24^

Radostits et al have reported that colibacillosis is the major cause of death in neonatal animals, because maternal immunoglobins are not transferred transplacentally.^25^ In addition, whereas castor oil obtained from *Ricinus communis* is used in the treatment of constipation, sometimes the oil causes diarrhea in both human and animals. Since a number of plants can be used to treat diarrhea induced by bacteria or toxicants,^26^, ^27^, ^28^, ^29^, ^30^ we investigated the effects of a root bark extract of *B. coccineus* on chemically and bacterially induced diarrhea.

## 2 MATERIALS AND METHODS

### 2.1 Sample collection and identification

Samples of the root, stem and leaves of *B. coccineus* were collected from Alkpali village in Ugbokolo, Okpokwu Local Government Area of Benue State, Nigeria. The plant parts were taken to the Department of Biological Science, Federal University of Agriculture, Makurdi for identification and confirmation by Mr Namadi Sanusi of Ahmadu Bello University Zaria Habarium and assigned Voucher number 900 237.

### 2.2 Extraction of *B. coccineus* root bark

About 1.5 kg of *B. coccineus* root was obtained, washed with distilled water and subsequently air dried in the laboratory at room temperature. The dry root bark was pulverized into fine powder using a grinder and kept in a cellophane bag at 4°C until use. Fifty grams (50 g) of *B. coccineus* root powder was placed in a conical flask containing 100 mL of distilled water. The mixture was thoroughly shaken intermittently throughout the period of extraction using a stirrer, allowed to stand overnight, filtered with Whatman No.1 filter paper into a measuring cylinder, concentrated at 60°C in an incubator and stored in a refrigerator at 4°C until required.^21^

### 2.3 Qualitative phytochemical analysis

The aqueous extract of *Brysocarpus coccineus* root bark was qualitatively evaluated for the presence of alkaloids, cardiac glycosides, saponins, tannins, steroids, flavonoids, anthraquinones, total glycosides, and reducing sugars using the methods of Trease and Evans,^31^ Harborne 32 and Edeoga et al.^33^

### 2.4 Quantitative phytochemical analysis 

The root bark powder of *Byrsocarpus coccineus* was quantitatively analyzed for the presence of alkaloid, flavonoid, phenol, saponin and tannin according to the methods described by Ordonez et al,^34^ Van Burden and Robinson^35^ and Skerget et al.^36^

### 2.5 Experimental animals

A total of one hundred and ten (110) adult Wistar albino rats of both sexes (55 males; 55 females), weighing 218 ± 28.6 g were used for the study. The animals were obtained from the National Veterinary Research Institute, Vom, Plateau State, Nigeria and were kept under standard housing conditions and allowed to acclimatize for 2 weeks before commencement of the study. They were fed with a commercial rat feed, Finisher^®^ produced by Grand Cereals and Oil Mills Ltd. Clean water was provided *ad libitium*. The research was carried out according to protocols and procedures approved by the Department of Veterinary Biochemistry, Physiology and Pharmacology Ethical Committee, Federal University of Agriculture Makurdi, Nigeria.

### 2.6 Acute oral toxicity study

The up and down procedure revised by Saganuwan^37^ was adopted for determination of median lethal dose (LD_50_) using five female rats. All rats were dosed at 5000 mg/kg body weight and observed for 48 hours in the following sequence. The first rat was dosed and observed for 48 hours. The second and third rats were then concurrently dosed and observed. The fourth and fifth rats were then sequentially dosed and observed. All the rats were observed for an additional 12 days for signs of delayed toxicity or death.

### 2.7 Effects of *B. coccineus* extract on castor oil‐induced diarrhea in rats

Twenty‐five female rats were used for this study. The method of Offiah and Chikwendu^38^ was adopted. The rats were starved overnight prior to the study, but allowed access to water. They were separated into five groups of five. The rats in groups I‐‐III were respectively orally administered 50, 100 and 200 mg/kg body weight of the extract, while the rats in group IV were administered 2 mL of normal saline. The group V rats were administered the antidiarrheal diphenoxylate hydrochloride (5 mg/kg) by intraperitoneal route. All the rats were housed singly in separate cages lined with white blotting paper. One hour after treatment, each of the rats was treated with 1 mL of castor oil, orally. The rats were then observed for 6 hours for watery faeces. All the observations were recorded as percentage (%) protection.

### 2.8 Effects of *B. coccineus* extract on gastrointestinal motility

The method of Chime et al^39^ was used to test the effect of the extract on gastrointestinal motility. Twenty‐five female rats were fasted overnight, with access to water. They were then divided into five groups of five. The rats in group I were orally administered 2 mL normal saline (0.9%), those in group II were administered 3 mg/kg of the antidiarrheal atropine intraperitoneally, and those in groups III‐‐V were orally administered 50, 100, and 200 mg/kg of the extract, respectively. One millilitre (1 mL) of castor oil was administered to each animal, before administration of the drug and the extract. Thirty minutes after the drug and extract administration, 1 mL of 5% activated charcoal suspension in a 10% aqueous solution of acacia powder was administered, and the rats were sacrificed 30 minutes later. The abdomens were opened and the distances travelled by the charcoal meal were measured and expressed as a percentage of the total length of the intestine from pylorus to caecum.^40^

### 2.9 Effects of *B. coccineus* extract on castor oil‐induced enteropooling

The intraluminal fluid accumulation due to the effect of castor oil was determined by the method of Robert et al^41^ Twenty‐five male rats, separated into five groups of five, were used for this study. The rats were fasted overnight, before the commencement of the study. Group I rats were orally administered 2 mL normal saline, group II rats were administered atropine (3 mg/kg) intraperitoneally, and rats in groups III‐‐V were orally administered 50, 100 and 200 mg/kg of the extract, respectively. After 1 hour, castor oil was administered to each animal. The animals were sacrificed immediately and the small intestine removed, tied on both ends with thread and weighed. The intestinal content was collected by milking and the quantity measured.

### 2.10 Determination of antibacterial activity of *B. coccineus* extract

To test the antibacterial activity of the *B. coccineus* extract, laboratory isolates of pure cultures of two gram‐positive species (*Streptococcus pyogenes* and *Enterococcus fecalis*) and three gram‐negative species (*S. pullorum, E. coli* and *Pseudomonas aeruginosa*) were obtained from the Department of Veterinary Pathology and Microbiology, Federal University of Agriculture, Makurdi, Benue State, Nigeria.

### 2.11 Determination of in vitro antibacterial activity

The well diffusion method described by the National Committee of Clinical Laboratory Standards^42^ was used to determine antibacterial activity. A Mcfarland standardized innoculum (0.1 mL) was spread on the surface of prepared nutrient agar plates using a glass spreader. Three wells of 3 mm in diameter were made per plate. Different concentrations (50, 100, and 200 mg/mL) of the extract solution were introduced into each well. The plates were incubated overnight at 37°C. For each isolate, a zone of inhibition above 6 mm in diameter was used as a measure of the susceptibility of the test organisms to the extract or a standard antibiotic (oxytetracycline).

### 2.12 Minimal inhibitory concentration

The minimal inhibitory concentration of the extract was determined using the dilution method described by Greenwood.^43^ Serial dilutions of the extract were carried out to obtain concentrations of 2.5, 1.25, 0.63, 0.31, 0.16, and 0.07 mg/mL. A drop of the test organism was added to each concentration and incubated at 37°C for 24 hours. Serial dilutions of the standard antibiotic (oxytetracycline) were made and tested for comparison.

### 2.13 In vivo antibacterial activity of *B. coccineus* extract in Wistar albino rats infected

#### 2.13.1 *Escherichia coli*

##### Preparation of *E. coli* inoculum

The pathogenic *E. coli* strain used for these tests was obtained from the Department of Veterinary Microbiology, Ahmadu Bello University, Zaria. The infective inoculum was prepared from an overnight nutrient broth culture of the isolate and the number of organisms estimated as colony forming units (CFU) per millitre of suspension in sterile nutrient broth, matched using a standard MacFarland scale. Colony count was performed on the diluted culture to achieve a titre of 3.0 × 10^6^ CFU/mL.^44^ All the rats were starved overnight, before the experimental infection. Each rat was inoculated orally with 0.5 mL of the inoculum. The uninfected control rats were inoculated with sterile broth.

##### Experimental groups and *E. coli* inoculation

Thirty male rats were randomly divided into six groups (I‐VI) of five and kept in separate cages. Groups I, II and III were inoculated with *E. coli* and treated with the extract at doses of 50, 100 and 200 mg/kg, respectively. Group IV was inoculated with *E. coli* and treated with 5 mg/kg of 5% oxyteyracycline (positive control), and group V was inoculated but untreated (negative control). Group VI served as the normal control group (not inoculated and non‐treated). Oxytetracycline (5 mg/kg, manufactured by Hebe Yuanzheng Pharmaceutical Limited) was used as the standard antibiotic. Treatment with the extract and standard antibiotic commenced 3 days post infection and lasted for a period of 7 days.

#### 2.13.2 Clinical signs and treatments

Rats from all the groups were closely observed for clinical signs of infection. The morbidity and mortality in all the groups was recorded. The body weights of the rats were recorded at days 3, 7, 12 and 18 post‐infection.

#### 2.13.3 Hematology

Blood samples obtained through the media canthus of the eye were used for the determination of hematological parameters such as red blood cell (RBC) count, packed cell volume (PCV), hemoglobin concentration and white blood cell (WBC) count.^45^

#### 2.13.4 Histopathological examination

Two rats randomly selected from each group, making a total of 12, were sacrificed using phenobarbitone (37 mg/kg) on day 19 post infection. The liver, kidney and intestine were collected from the rats and fixed in Bouin's fluid for 24 hours. The tissues were dehydrated through graded concentrations of ethanol (70%, 95% and 100%), cleared in xylene and embedded in paraffin wax. The embedded tissues were sectioned at 7 μm thickness and stained with Hematoxylin and Eosin (H&E) for light microscopic examination. Photomicrographs of the sections were taken using a digital camera.^46^

### 2.14 Statistical analysis

The results of the quantitative analysis of phytochemical contents are presented as percentiles. The frequencies of droppings, gastrointestinal transit time, enteropooling, antimicrobial zones of inhibition and average organ weight gain were calculated using one‐way analysis of variance. The least significance difference was set at the 5% level. The minimum inhibitory concentration of the test microorganism, average weight gains of the infected rats, RBC, PCV and WBC counts, hemoglobin, total blood volume, red cell volume, and plasma volume were calculated using two‐way analysis of variance. Fisher's test was used to detect significant differences, set at the 5% level.^47^

## 3 RESULTS

### 3.1 Qualitative phytochemical analysis

Results of the qualitative phytochemical analysis of the aqueous root bark extract of *B. coccineus* revealed the presence of flavonoids, tannins, saponins, alkaloids and phenols. The analysis also revealed the absence of steroids, terpenoids, cardiac glycosides and anthroquinones (Table 1).

**Table 1:** The phytochemical constituents of an aqueous root bark extract of *Byrsocarpus coccineus*

Phytochemical constituents	Results
Flavonoids	+
Tannins	+
Saponins	+
Alkaloids	+
Phenol	+
Carbohydrate	+
Cardiac glycoside	−
Terpernoid/ steroid	−
Anthraquinone	−

Note

+ = presence; − = absence.

### 3.2 Quantitative phytochemical analysis

This analysis showed the quantity of various constituents present in the aqueous root bark extract of *B. coccineus.* Saponins were present at the highest quantity (10.6%), followed by tannins (7.6%), flavonoids (6.2%), phenols (5.8%) and alkaloids (4.4%) (Table 2).

**Table 2:** The quantitative analysis of the phytochemical content of the aqueous root bark extract of *Byrsocarpus coccineus*

Phytochemical	Alkaloids	Saponins	Flavonoids	Tannins	Phenols
Percentage present (%)	4.4	10.6	6.2	7.6	5.8

### 3.3 Acute oral toxicity study

Oral administration of the aqueous root bark extract of *B. coccineus* to rats produced no visible signs of toxicity at 5000 mg/kg body weight. Hence, the median lethal dose (LD_50_) value for the extract was estimated to be above 5000 mg/kg.

### 3.4 Effects of *B. coccineus* extract on castor oil‐induced diarrhea

Table 3 shows the frequency of defecation of the rats within 6 hours of administration of the extract and castor oil. There was a significant difference (*P* < .05) in the frequency of defecation between the control group and the treatment groups. The group treated with 100 mg/kg had the lowest frequency of defecation and highest percentage inhibition (74.96%). There was no significant difference in the percentage inhibition between the groups administered aqueous extract of *B. coccineus* at different doses and the group treated with diphenoxylate at 5 mg/kg body weight.

**Table 3:** The effect of the aqueous root bark extract of *Byrsocarpus coccineus* on castor oil‐induced diarrhea in Wistar albino rats

S/No.	Group	Treatment (mg/kg)	No. of defecations (6 h)	Percentage protection
I	Control	Normal saline	6.67 ± 1.45	—
II	Diphenoxylate + CO	5	2.33 ± 0.33	65.06
III	Extract + CO	50	2.00 ± 1.00^a^	70.01^a^
IV	Extract + CO	100	1.67 ± 0.88^a^	74.96^a^
V	Extract + CO	200	2.33 ± 1.45	65.06

Abbreviation: CO, castor oil.

a Significant at *P* < .05 compared to the control. Values are presented as means ± SEM based on 5 observations.

### 3.5 Effects of *B. coccineus* extract on gastrointestinal transit of charcoal

The effect of the extract on gastrointestinal transit of charcoal is shown in Table 4. There was a significant (*P* < .05) decrease in the intestinal transit of charcoal in the extract‐treated groups compared to the control group. The charcoal travelled very rapidly along the intestine in the control group, while the rate of movement was significantly (*P* < .05) reduced in the rats treated with the extract. The rats treated with 3 mg/kg of atropine had a slower rate of charcoal movement along the small intestine compared to the extract‐treated rats (Table 4). The transit of charcoal in the groups treated with the extract appeared to be statistically similar to the group treated with atropine.

**Table 4:** Effect of the aqueous root bark extract of *Byrsocarpus coccineus* on gastrointestinal transit of charcoal in Wistar albino rats

S/No.	Treatment group	Length of intestine (cm)	Distance travelled by charcoal (cm)	%Intestinal transit
I	Control	37.3 ± 2.2	29.0 ± 2.1	77.75
II	Atropine (3 mg/kg)	38.3 ± 1.3	14.7 ± 3.9^a^	38.38
III	Extract (50 mg/kg)	40.0 ± 2.0	16.3 ± 2.7^a^	40.75
IV	Extract (100 mg/kg)	38.7 ± 3.2	16.7 ± 1.5^a^	43.15
V	Extract (200 mg/kg)	38.7 ± 1.5	20.7 ± 1.5^a^	53.49

a Significant at *P* < .05 compared to the control. Values are means ± SEM based on 5 observations.

### 3.6 Effects of *B. coccineus* extract on castor oil‐induced enteropooling

The effect of the extract on castor oil‐induced enteropooling is shown in Table 5. The results showed that there was a significant difference (*P < *.05) in the quantity of intestinal contents between the control and the treated groups. The amounts of fluid in the extract‐treated groups were similar to that of atropine‐treated group. The atropine‐treated group had the highest percentage of intestinal fluid inhibition at 36.0%, followed by the group treated with 200 mg/kg of the extract (21.74%), while the groups treated with 50 mg/kg and 100 mg/kg had the lowest percentage intestinal fluid inhibition (14.71%) compared with the standard drug (atropine sulphate).

**Table 5:** Effect of the aqueous root bark extract of *Byrsocarpus coccineus* on castor oil‐induced enteropooling in rats

S/No.	Treatment	Weight of full intestine (g)	Weight of empty intestine (g)	Weight of intestinal content (g)	%Inhibition
I	Control + CO	4.69 ± 0.33	2.52 ± 0.00	2.17 ± 0.67	12.84
II	Atropine (3 mg/kg + CO	3.00 ± 0.00	1.33 ± 0.33	1.67 ± 0.33^a^	36.0
III	Extract (50 mg/kg) + CO	4.00 ± 0.57	2.67 ± 0.67	1.33 ± 0.33^a^	14.71
IV	Extract 100 mg/kg) + CO	4.00 ± 0.67	2.67 ± 0.67	1.67 ± 0.67^a^	14.71
V	Extract + CO (200 mg/kg)	3.67 ± 0.33	2.33 ± 0.33	1.00 ± 0.58^a^	21.74

Abbreviation: CO, castor oil.

a Significant at *P* < .05 compared to the control. Values are means ± SEM based on 5 observations.

### 3.7 Antibacterial effects of *B. coccineus* aqueous root bark extract

At the various concentrations used, *E. coli* and *S. pullorum* were susceptible to the aqueous root bark extract of *B. coccineus*, while *Streptococcus pyogenes, Enterococcus fecalis and Psuedomonas aeruginosa* were resistant to the aqueous extract at the various concentrations used (Table 6). The activity of the extract was dose dependent, with the highest activity recorded at 400 mg/mL for *E. coli* (zone of inhibition 3.7 ± 0.2 mm^2^) and *S.* pullorum (3.6 ± 0.7 mm^2^). The minimum inhibitory concentration of the extract recorded was 0.31 mg/mL for *E. coli* (0.7 ± 0.1 mm^2^) and the greatest effects were seen at a concentration of 2.5 mg/mL for *E. coli* (1.05 ± 0.4 mm^2^) and *S.* pullorum (1.1 ± 0.3 mm^2^) (Table 7)*.*

**Table 6:** In vitro antimicrobial activities of higher doses of the aqueous root bark extract of *Byrsocarpus coccineus* without control

Extract concentration (mg/mL)	*E. coli*	*S. pullorum*	*S. pyrogenes*	*P. aeruginosa*	*E. fecalis*
100	2.5 ± 0.1	2.0 ± 0.4	0.00 ± 0.00	0.00 ± 0.00	0.00 ± 0.00
200	3.1 ± 0.4	3.03 ± 0.1	0.00 ± 0.00	0.00 ± 0.00	0.00 ± 0.00
400	3.7 ± 0.2	3.6 ± 0.7	0.00 ± 0.00	0.00 ± 0.00	0.00 ± 0.00

Note

Values are means ± SEM based on 3 observations.

**Table 7:** In vitro inhibitory effect of low doses of aqueous root bark extract of *Byrsocarpus coccineus*

	*E. coli*	*S. pullorum*	*S. pyrogenes*	*P. aeruginosa*	*E. species*
Extract concentration (mg/mL)					
2.5	1.05 ± 0.4	1.1 ± 0.3	0.0 ± 0.0	0.0 ± 0.0	0.0 ± 0.0
1.25	1.2 ± 0.1	1.3 ± 0.1	0.0 ± 0.0	0.0 ± 0.0	0.0 ± 0.0
0.63	1.0 ± 0.1	1.0 ± 0.4	0.0 ± 0.0	0.0 ± 0.0	0.0 ± 0.0
0.31	0.7 ± 0.1	0.9 ± 0.3	0.0 ± 0.0	0.0 ± 0.0	0.0 ± 0.0
0.16	0.0 ± 0.0	0.0 ± 0.0	0.0 ± 0.0	0.0 ± 0.0	0.0 ± 0.0
0.07	0.0 ± 0.0	0.0 ± 0.0	0.0 ± 0.0	0.0 ± 0.0	0.0 ± 0.0
Conc. of control (mg/mL)					
2.5	2.7 ± 0.1	0.8 ± 0.1		3.4 ± 0.4	2.6 ± 0.1
1.25	2.6 ± 0.1	0.8 ± 0.1		3.3 ± 0.2	2.2 ± 0.1
0.63	2.6 ± 0.2	0.3 ± 0.2		2.6 ± 0.1	2.3 ± 0.1
0.31	2.4 ± 0.1	0.0 ± 0.0		2.3 ± 0.1	2.0 ± 0.1
0.16	2.0 ± 0.1	0.0 ± 0.0		2.0 ± 0.1	1.9 ± 0.1
0.07	2.0 ± 0.1	0.0 ± 0.0		1.5 ± 0.1	1.5 ± 0.1

Note

Values are means ± SEM based on 3 observations.

### 3.8 In vivo antibacterial effects of *B. coccineus *extract on Wistar albino rats infected with *E. coli*

#### 3.8.1 Clinical signs

Rats from infected groups showed signs of depression and anorexia at day 3 post infection, and by day 4 post infection there was diarrhea accompanied by weakness in 50%‐60% of the infected groups. Treatment with the extract resulted in decreased severity of the clinical signs, particularly diarrhea at day 2 of the treatment (ie day 5 post infection). The observed clinical signs persisted in the infected untreated group up to day 18 post infection, when the study was terminated.

#### 3.8.2 Effects of *E. coli* infection and the extract treatment on body weight gain of rats

Table 8 showed the results of the various treatments on the body weight gain of rats infected with *E. coli*. The infection caused a reduction in the body weight gain of infected rats compared with the control group. The extract caused an improvement in the body weight gain compared to the infected untreated group. There was a significant (*P* < .05) decrease in the body weights of the extract‐treated groups compared to those of oxytetracycline‐treated and uninfected control groups. The body weights of the rats in the infected untreated group were the lowest among all the groups.

**Table 8:** Effect of aqueous root bark extract of *Byrsocarpus coccineus* on body weights of rats infected with *E. coli*

S/No.	Treatment/group	Day 3	Day 7	Day 12	Day 18
I	50 mg/kg	129.11 ± 5.29	148.54 ± 11.77	158.41 ± 12.68	159.00 ± 12.32
II	100 mg/kg	122.71 ± 5.27	147.75 ± 11.30	155.74 ± 10.15	154.19 ± 11.21
III	200 mg/kg	125.26 ± 10.51	140.62 ± 5.23	144.16 ± 4.25	143.08 ± 2.89
IV	5 mg/kg (5% oxytetracydine)	192.55 ± 9.57	198.31 ± 5.51	204.25 ± 5.72	205.74 ± 5.57
V	Infected untreated	114.14 ± 11.76	128.49 ± 10.95	131.52 ± 11.44	133.84 ± 11.02
VI	Uninfected untreated	216.03 ± 11.39	219.08 ± 8.36	221.98 ± 8.36	221.76 ± 7.95

Note

Values are means ± SEM based on 5 observations.

Table 9 showed the effect of aqueous root bark extract of *B. coccineus* on the liver‐, left kidney‐ and right kidney‐to‐body weight ratio of rats infected with *E. coli*. There was significant increased weight ratio (*P* < .05) for liver, left kidney and right kidney at 50, 100 and 200 mg/kg of the extract. The body weight gain decreased in the dose range of 50‐‐200 mg/kg of the extract, but the weight of the infected untreated group decreased significantly (*P* < .05) (Table 9).

**Table 9:** Effects of the aqueous root bark extract of *Byrsocarpus coccineus* on the weight of liver‐ and kidney‐to‐body weight ratio of rats infected with *E. coli*

S/No.	Group	Body weight	Liver	Left kidney	Right kidney
I	50 mg/kg	148.77 ± 13.95^a^	0.04 + 0.02^a^, ^a^	0.04 ± 0.001^a^, ^a^	0.004 ± 0.001^a^, ^a^
II	100 mg/kg	145.10 ± 15.32^a^	0.05 ± 0.03^a^, ^a^	0.04 ± 0.001^a^, ^a^	0.004 ± 0.004
III	200 mg/kg	138.28 ± 8.81^a^	0.04 ± 0.01^a^, ^a^	0.01 ± 0.01	0.004 ± 0.001^a^, ^a^
IV	5 mg/kg (5% oxytetracycline.)	200.21 ± 6.03	0.04 ± 0.04	0.004 ± 0.003	0.004 ± 0.004
V	Infected untreated	126.00 ± 8.85^a^	0.05 ± 0.05	0.004 ± 0.003	0.004 ± 0.003
VI	Uninfected untreated	219.71 ± 2.79	0.03 ± 0.03	0.003 ± 0.003	0.003 ± 0.003

a Significant at *P* < .05 compared to the controls. Values are means ± SEM based on 5 observations.

#### 3.8.3 Effects of *E. coli* infection and the extract treatment on hematological parameters of rats

The effects of *E. coli* infection and the various treatments on the hematological parameters of infected rats are presented in Tables 10‐14. Infection of rats with *E. coli* decreased the red blood cells on day 7 post infection (Table 10). Treatment with the extract and oxytetracycline on day 8 improved the RBC values of the treated animals on day 18 post infection (ie 10 days post treatment).

**Table 10:** Effects of the aqueous root bark extract of *Byrsocarpus coccineus* on mean red blood cell count (× 10^6^/mm^3^) of rats infected with *E. coli*

S/No.	Treatment group	Day 3	Day 7	Day 18
I	50 mg/kg	6.36 ± 0.47	3.42 ± 1.46^a^	5.75 ± 0.32
II	100 mg/kg	6.38 ± 0.87^a^	6.57 ± 1.61	6.85 ± 0.29
III	200 mg/kg	6.08 ± 0.36	4.65 ± 1.94	7.24 ± 0.29
IV	5 mg/kg (5% oxtetracycline)	6.67 ± 0.32	5.70 ± 0.61^a^	6.34 ± 0.36
V	Infected not treated	6.88 ± 0.72	1.70 ± 0.47^a^	5.99 ± 0.44
VI	Uninfected untreated	6.09 ± 0.15	6.06 ± 0.14	4.87 ± 1.37

a Significant at *P* < .05 compared to the controls. Values are means ± SEM based on 5 observations.

Infection of rats with *E. coli* resulted in a decreased packed cell volume (Table 11). The highest decrease occurred in the infected untreated group. The PCV values of the groups treated with the extract were higher than that of the infected untreated group on day 7 post infection (ie 4 days of treatment). Thereafter, the PCV values improved to reach the highest level on day 18 post infection in the group treated with 200 mg/kg of the extract. The effect of aqueous root bark extract of *B. coccineus* on the hemoglobin concentration of the rats infected with *E. coli* is shown in Table 12. On day 3 post infection, the hemoglobin values of the infected groups were not significantly (*P* > .05) different from that of the uninfected control, except in the group treated with 100 mg/kg of the extract, which had a hemoglobin value of 10.09 ± 2.06 g/L. However, on day 7 post infection, the hemoglobin value of the group infected and treated with 100 mg/kg of the extract improved and was significantly (*P* < .05) higher than that of the uninfected untreated control. On day 18 post infection (ie 10 days post infection), the infected group treated with 50 mg/kg had the lowest hemoglobin value of 11.70 ± 0.21 g/L.

**Table 11:** Effects of the aqueous root bark extract of *Byrsocarpus coccineus* on packed cell volume (%) of rats infected with *E. coli*

S/No.	Treatment (mg/kg)	Day 3	Day 7	Day 18
I	50 mg/kg	43.53 ± 1.32^a^	23.00 ± 9.75	32.83 ± 3.32
II	100 mg/kg	43.49 ± 1.31^a^	40.70 ± 9.56	35.38 ± 1.22
III	200 mg/kg	41.93 ± 2.02^a^	29.57 ± 11.86	41.42 ± 0.93
IV	5 mg/kg (5% oxtetracycline)	46.38 ± 0.38^a^	19.47 ± 4.37	37.50 ± 0.74
V	Infected not treated	46.52 ± 1.64	12.22 ± 3.11	34.30 ± 1.30
VI	Not infected not treated	47.68 ± 1.23	47.67 ± 1.21	47.33 ± 1.34

a Significant at *P* < .05 compared to the controls. Values are means ± SEM based on 5 observations.

**Table 12:** Effects of the aqueous root bark extract of *Byrsocarpus coccineus* on white blood cells (WBC) of rats infected with *E. coli*

S/No.	Treatment (mg/kg)	Day 3	Day 7	Day 18
I	50 mg/kg	8.40 ± 0.85^a^	11.05 ± 0.69^a^	7.33 ± 0.89^a^
II	100 mg/kg	5.65 ± 097^a^, ^b^	10.82 ± 2.49^a^	6.87 ± 1.09^a^
III	200 mg/kg	7.73 ± 1.56^a^	10.87 ± 2.13^a^	8.80 ± 0.52^a^
IV	5 mg/kg (5% oxtetracycline)	9.48 ± 0.68^a^, ^b^	8.70 ± 1.00	6.90 ± 0.74
V	Infected untreated	10.87 ± 2.15	8.22 ± 1.30	7.93 ± 1.43
VI	Uninfected untreated	6.05 ± 0.70	7.25 ± 1.81	6.53 ± 1.93

Note

Values are mean ± SEM base on 5 observations

a Significantly lower at *P* < .05 compared to the controls.

b Significantly lower at *P* < .05 compared to infected untreated group.

The lowest white blood cell count ((5.65 ± 0.97) × 10^9^) was recorded on day 3 post infection in the group infected with *E. coli* and treated with 100 mg/kg of the extract, while the infected untreated group had the highest WBC value ((10. 87 ± 2.15) × 10^9^). The WBC values of the *E. coli* infected groups treated with the varying extract doses were significantly higher than that of the uninfected untreated control group on day 7 post infection. The increased values in the extract‐treated groups later decreased by day 18 post infection, and the values appear to be similar to that of the uninfected control group (Table 13). There were significant (*P* < .05) increases in neutrophil counts in all the infected groups treated with the extract on day 3 post infection. Treatment with the extract decreased the neutrophil values below that of the group infected and treated with oxytetracycline and the group infected but not treated, on day 7 post infections. All the infected groups had significantly (*P* < .05) increased neutrophil counts on day 18 compared to the uninfected control group. Basophils, eosinophils and monocytes were increased significantly (*P* < .05) at various doses on days 3, 7 and 18 post‐infection (Table 13).

**Table 13:** Effects of the aqueous root bark extract of *Byrsocarpus coccineus* on differential white blood cell counts of rats infected with *E. coli*

Parameter	Treatment groups (mg/kg)	Day 3	Day 7	Day 18
Basophils	50 mg/kg	0.16 ± 0.03^a^	0.12 ± 0.02	0.13 ± 0.01^a^
	100 mg/kg	0.16 ± 0.04^a^	0.04 ± 0.02	0.11 ± 0.02
	200 mg/kg	0.17 ± 0.01^a^	0.13 ± 0.01	0.11 ± 0.01
	5 mg/kg (5% oxtetracycline)	0.13 ± 0.02	0.15 ± 0.01	0.10 ± 0.01
	Infected untreated	0.12 ± 0.02	0.16 ± 0.03	0.12 ± 0.02
	Uninfected untreated	0.14 ± 0.03	0.09 ± 0.01	0.10 ± 0.03
Eosinophils	50 mg/kg	0.44 ± 0.09^a^	0.31 ± 0.06^a^	0.35 ± 0.02^a^
	100 mg/kg	0.43 ± 0.10^a^	0.11 ± 0.04^a^	0.28 ± 0.04^a^
	200 mg/kg	0.44 ± 0.03^a^	0.34 ± 0.03^a^	0.29 ± 0.04^a^
	5 mg/kg (5% oxtetracycline)	0.34 ± 0.04	0.41 ± 0.04^a^	0.28 ± 0.03^a^
	Infected untreated	0.33 ± 0.05	0.44 ± 0.09^a^	0.32 ± 0.06
	Uninfected untreated	0.37 ± 0.07	0.25 ± 0.02	0.26 ± 0.06
Neutrophils	50 mg/kg	5.44 ± 1.07^a^	3.93 ± 0.57^b^	3.94 ± 0.57^a^
	100 mg/kg	5.41 ± 1.23^a^	3.86 ± 0.78^b^	3.44 ± 0.54^a^
	200 mg/kg	5.53 ± 0.35^a^	1.33 ± 0.49^b^	3.66 ± 0.45^a^
	5 mg/kg (5% oxtetracycline)	4.18 ± 0.70	4.20 ± 0.42^a^	3.45 ± 0.37^a^
	Infected untreated	4.11 ± 0.66	4.85 ± 0.45^a^	5.96 ± 0.72^a^
	Uninfected untreated	4.63 ± 0.90	3.01 ± 1.78	3.26 ± 0.97
Monocytes	50 mg/kg	0.54 ± 0.11^a^	0.39 ± 0.08	0.44 ± 0.03^a^
	100 mg/kg	0.54 ± 0.13^a^	0.13 ± 0.05	0.34 ± 0.05
	200 mg/kg	0.55 ± 0.04^a^	0.42 ± 0.04	0.36 ± 0.04^a^
	5 mg/kg (5% oxtetracycline)	0.44 ± 0.05	0.49 ± 0.05	0.35 ± 0.04^a^
	Infected untreated	0.51 ± 0.12	0.54 ± 0.11	0.40 ± 0.07
	Uninfected untreated	0.46 ± 0.09	0.31 ± 0.03	0.33 ± 0.10

a Significant at *P* < .05 compared to the controls.

b Significantly lower at *P* < .05 compared to the controls. Values are means ± SEM based on 5 observations.

### 3.9 Histopathological changes

**Figure 1:**
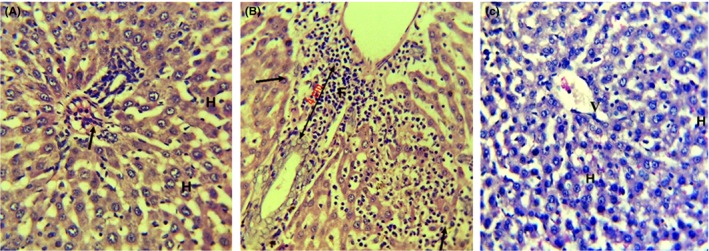
Photomicrograph of the liver of a Wistar albino rat. Uninfected untreated (A), infected untreated (B), infected and treated with 200 mg/kg of aqueous root bark extract of *Byrsocarpus coccineus* (C) (H&E, ×400)

**Figure 2:**
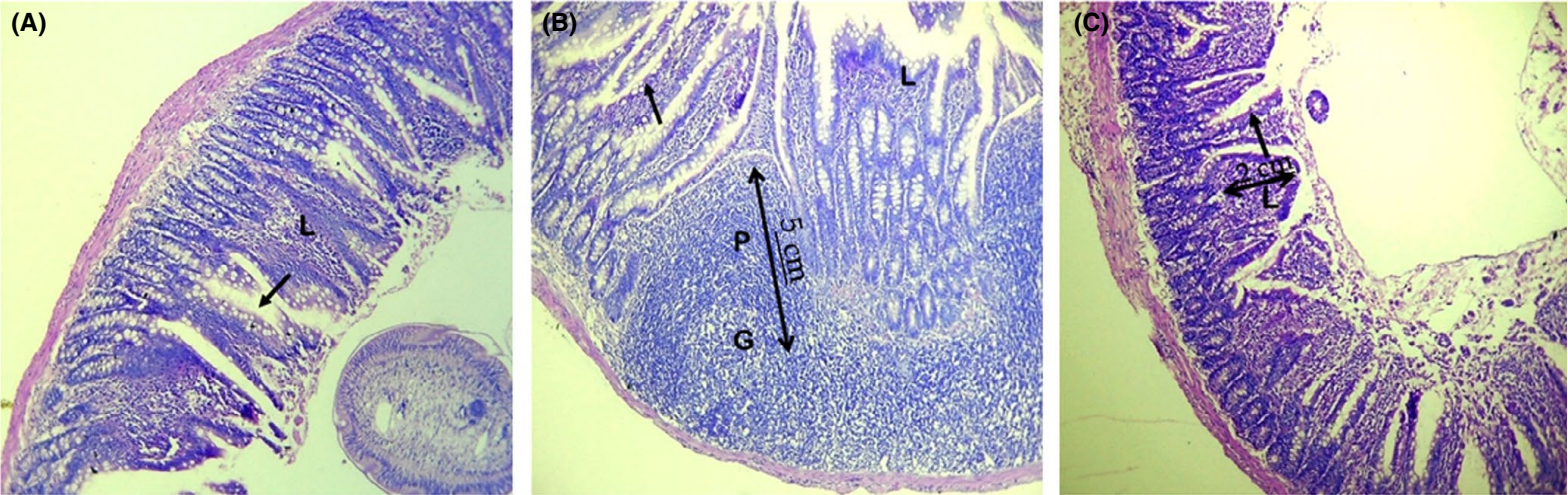
Photomicrograph of the small intestine of a Wistar albino rat. Uninfected untreated (A), infected untreated (B), infected and treated with 100 mg/kg of aqueous root bark extract of *Byrsocarpus coccineus* (C) (H&E, ×400)

A photomicrograph of the normal liver of a Wistar albino rat (uninfected and untreated) showed a normal portal triad (arrow) and hepatocytes (H) (Figure 1A). However, a photomicrograph of the liver of a Wistar albino rat infected with *E. coli* but neither treated with extract nor the antibiotic showing marked cellular infiltration around the portal triad (F) and between the hepatocytes (arrow) (Figure 1B). The photomicrograph of the liver of a Wistar albino rat infected with *E. coli* and treated with 200 mg/kg of the aqueous root bark extract of *B. coccineus* showed a normal central vein (V) and hepatocytes (H) (Figure 1C). In the intestine, there was severe villous collapse, with matting and fusion of the villi (Figure 2A).

Figure 2B is a photomicrograph of the small intestine of a Wistar albino rat infected with *E. coli* and not treated, showing reactive Peyer's patches (P) with a germinal centre (G) and numerous goblets cells (arrow) along the walls of the villi and mild intraepithelial cellular infiltration (L). The small intestine of a Wistar albino rat infected with *E. coli* and treated with 100 mg/kg of the aqueous root bark extract of *B. coccineus* showed numerous goblet cells (arrow) along the walls of the villi and mild intraepithelial cellular infiltration (L) (Figure 2C).

**Figure 3:**
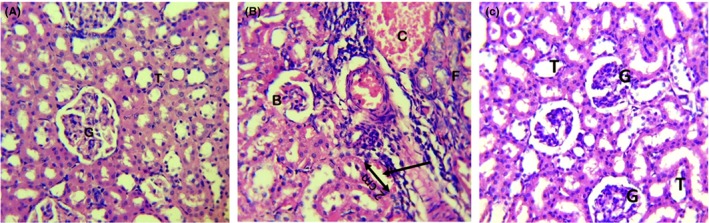
Photomicrograph of the kidney of a Wistar albino rat. Uninfected untreated (A), infected untreated (B), infected and treated with 200 mg/kg of aqueous root bark extract of *Byrsocarpus coccineus* (C) (H&E, ×400)

In the kidney, there was marked congestion, shrunken glomeruli with a wide bowman capsule, tubular necrosis and cellular infiltration in rats infected with *E. coli* but not treated (Figure 3B). The kidney of the infected rats had severe lesions. The rats treated with 200 mg/kg of the extract showed evidence of recovery (Figure 3C). The kidney from the control was normal (Figure 3A). Generally, the extract‐treated groups had less severe lesions compared with the infected untreated groups.

## 4 DISCUSSION

The use of plants and plant products in the treatment of diseases and other health problems can be attributed to the presence of phytochemical compounds with varying pharmacological and medicinal values. Many of these plants are known to possess both pharmacological and physiological activities.^48^, ^49^ Phytochemical analysis is a very significant aspect of herbal or medicinal plant research, and is used to evaluate the presence of chemical constituents with medicinal value in plants.^49^ Flavonoids present in the extract have been reported to possess antioxidant properties.^50^ Quercetin, kaempferol and quercitrin are flavonoids commonly present in plants. Tannins have astringent properties, which hasten wound healing and reduce mucus production in inflamed tissue.^51^ This activity is due to their ability to precipitate proteins, thereby protecting underlying tissues. They also inhibit microbial growth and proliferation.^52^

Saponins have been shown to possess hypolipidemic, anticancer, expectorant activities. They are used in the treatment of upper respiratory tract inflammation.^31^ Volatile oils (essential oils) contribute greatly as an essence in enhancing the aroma of some plant species.^53^ They are also used for the management of digestive, respiratory and urinary tract infections.^54^ Alkaloids are the largest group of secondary chemical constituents in plants, containing nitrogen‐based compounds synthesized from amino acids. They combine with acids to form salts.^55^ Therapeutically, alkaloids can serve as central nervous system (CNS) stimulants, anaesthetics, sedatives, analgesics, anti‐inflammatory agents, anticancer and muscle relaxants.^56^

Acute toxicity studies of extracts of *B. coccineus* showed that an oral LD_50_ > 5000 mg/kg is relatively safe,^57^, ^58^ indicating that the extract could be administered with some degree of safety at lower concentrations, especially via the oral route, where absorption may not be complete due to inherent factors limiting absorption in the gastrointestinal tract.^59^

Diarrhea involves frequent passage of unformed faeces at a rate higher than normal.^60^ Antidiarrheal agents are therefore required to restore normal stool frequency. In this study, the aqueous root bark extract of *B. coccineus* significantly reduced the diarrhea induced by castor oil. This was evident from its inhibitory effect on gastrointestinal tract, seen as a decrease in the peristaltic movement of a charcoal meal through the small intestine. This agrees with the report of Akindele and Adeyemi^22^ indicating that an aqueous leaf extract of *B. coccineus* significantly reduced gastrointestinal propulsion and secretion in castor oil‐induced diarrhea in mice and rats, and suggests that *B. coccineus* root bark possesses similar properties to the leaf. There was also a reduction in fluid secretion by the intestinal tract, as observed from the effects of the extract on castor oil‐induced enteropooling activity. This effect was similar to that of atropine, an antimuscarinic agent used in this study as a control. The antidiarrheal activity of the extract may be due to the presence of alkaloids in the plant, as atropine is also a plant alkaloid obtained from *Atropa belladonna*. Scopolamine is another alkaloid derived from *Hyoscyamus niger*, used as an antispasmodic in gastrointestinal disorders characterized by spasms.^61^ The percentage inhibition values for frequency of defecation following treatment with atropine, or increasing doses of extract were 65.0%, 70.01%, 74.96%, and 65.06%, respectively (Table 3).

The ricinoleic acid liberated from castor oil caused irritation of the gastrointestinal tract mucosa, resulting in inflammation, increased gastrointestinal secretion and enhanced motility of the gastrointestinal tract,^62^ leading to castor oil‐induced diarrhea.^1^ The diarrhea also may result from decreased absorption of substances within the intestine.^63^ Since the extract was able to inhibit castor oil‐induced diarrhea, its antidiarrhea effect may partly be due to its inhibitory effect on gastrointestinal secretion and/or gastrointestinal motility. The reduction in castor oil‐induced enteropooling may be due to the ability of the extract to reduce or prevent fluid and electrolyte secretion into the intestine, consequently decreasing gastrointestinal motility similarly to diphenoxylate, a synthetic piperidine opioid structurally related to pethidine, used clinically alone or in combination with atropine for control of non‐specific diarrhea in small animals.^5^, ^9^ Therefore, it could be inferred from the results of this study that the decreases in frequency of defecation and distance travelled by the charcoal meal may be due to the inhibition of gastrointestinal motility by the extract. It is possible that the effect of the extract may be mediated via α2 adrenergic or muscarinic receptor stimulation.

Our in vitro study of the effects of *B. coccineus* on *E. coli*, *S. pollorum*, *Enterococcus fecalis*, *Streptococcus pyogenes* and *Pseudomonas aeruginosa* showed that the extract is effective against *E. coli* and *S. pullorum* at the lowest concentration used, 0.31 mg/mL, whereas, *S. pyogenes*, *P. aeruginosa* and *E. fecali* were resistant to the extract. The antimicrobial activity of the extract shown here agrees with reports by Saganuwan and Gulumbe 26, 27, 28, 29, 30, 64, 65 indicating that medicinal plants such as *Abrus precatorius*, *Cassia occidentalis*, *Sida acuta subspecies acuta*, *Vernonia amygdalina* and *Ipomea sarifolia* have activity against *E. coli* and *S. typhimurium*. The phytochemicals responsible are alkaloids, saponins, tannins, glycosides and flavonoids. Therefore, the reported phytochemicals in the present study may be responsible for the antimicrobial activity of *Byrocarpus coccineus* extract. This suggests that the extract can be used to treat diarrhea caused by *E. coli* and *S. pullorum*, but will have no activity against diarrhea or infections caused by *Enterococcus fecalis, S. pyogenes* and *P. aeruginosa*, which are known to produce enterotoxins that damage the lining of the gastrointestinal mucosa, resulting in diarrhea.^66^ The susceptibility of *E. coli* and *S. pullorum* to the extract could be attributed to the presence of tannins and saponins observed to be present in the extract. Generally, tannin is known for its antimicrobial activities in the bark of plants where it protects the plant against bacteria and fungi infections. It also has soothing, anti‐inflammatory, antidiuretic and astringent activities.^67^ Significant beneficial effects attributed to the use of tannins are seen in wound healing, bleeding, tissue injury and skin regeneration. However, high tannin consumption has been implicated in osteoporosis and anemia in animals and humans due to its effects on calcium and iron absorption.^68^ Prohp and Onoagbe reported a tannin content of 12% in *Triplochitin scleroxylon* stem bark extract,^69^ hence the quantity of tannin (7.6%) obtained in this study may be considered safe.

The improved weight gain ratio and hematological parameters observed in the present study agree with the report of Saganuwan and Onyeyili^15^ indicating that medicinal plants can have positive effects on weight gain, hemoglobin content and plasma expansion. However, the body weights of the rats decreased with extract doses in the range of 50‐200 mg/kg body weight, suggesting that a decrease in body weight may not affect liver and kidney weight gain. The decreased body weight of the *E. coli‐*infected rats agrees with reports indicating that parasite infection can cause weight loss.^70^, ^71^ Body weight estimation is an index for measuring toxicities in animals.^72^ Saganuwan reported a significant number of medicinal plants that could be used in the treatment of diarrhea caused by chemical and infectious agents.^65^ Examples of such plants include *Anacardium occidentale*, *Psidium guajava*, *Sclerocarya birrea*, *Calotropis procera*, *Adansonia digitata*, *Anogeissus schimperi*, and *Capsicum ftutescens*, among others.^65^ The pathogenicity of the *E. coli* strain used in this study was confirmed based on the clinical symptoms, mortality and lesions noticed in the infected rats during the experimental period. The present findings agree with the report indicating that *E. coli* is a commensal bacterium of the intestine that is pathogenic to mammals.^73^ The main symptoms observed in *E. coli‐*infected rats, are not exclusive to this species;^74^, ^75^ *E. coli* strains associated with avian infection usually induce respiratory and septicaemic diseases.^76^

The efficacy of the extract in controlling the *E. coli* infection induced in this study may be due to the presence of some of the phytochemical compounds in the extract such as tannins, phenols, alkaloids and flavonoids,^77^ which are known to possess appreciable antibacterial activities. Tannins are known to decrease bacteria cell proliferation by blocking the enzymes of microbial metabolism, producing hydrogen bonds with the carbonyl group in the enzyme (redox reaction). An antimicrobial activity of flavonoids has also been demonstrated.^78^ The presence of flavonoids in the extract may thus be responsible for the significant neutrophil counts observed in the present study, since flavonoids are reported to have profound effects on the function of immune and inflammatory cells.^67^ Neutrophils play an important role in the defence mechanism of the body by providing the first line of defense against the invading microorganism, being free wandering cells in the body.^79^ The eosinophilia observed may be connected with inflammation of the kidney and liver.^80^ Eosinophils kill parasites, regulate the hypersensitivity reaction mediated by TgE antibodies and may promote inflammation and tissue damage.^81^ The general leucocytosis observed in the *E. coli‐*infected rats may be due to neutrophilia, eosinophilia, basophilia and monocytosis, as proven by differential leucocyte counts. Parasitic infections may be responsible for increased leucocyte counts.^70^ Hence the extract may be effective in the management of the clinical signs of *E. coli* infection. The in vivo control of *E. coli* replication by the extract demonstrated in this study is of immense clinical importance, since the development of resistance constitutes a major problem in the treatment and control of *E. coli* infection in both animals and man.

## 5 CONCLUSION

The *B. coccineus* extract tested in this study contains flavonoids, tannins, saponins, alkaloids, and phenols, which have potent antimicrobial activities against *E. coli* and *S. pollorum*, and as such may be used in the treatment of bacterially and chemically induced diarrhea in rats. The extract of this plant is apparently safe when administered orally. Hence the results of this study confirm folkloric claims that the aqueous root bark extract of *B.* *coccineus* can be used to prevent chemical and bacterial diarrhea. The root bark extract has hematinic and plasma expander effects in rats and may therefore be used in treatment of anaemia. It also has cellular immunostimulatory, hepatoprotective and anti‐inflammatory effects.

## CONFLICT OF INTEREST

None.

## AUTHOR CONTRIBUTIONS

EAS carried out the study, whereas PAO and SAS designed the study, analyzed the data, wrote and proofread the manuscript. SAS edited the manuscript finally. All authors approved the final version of the manuscript for submission.
